# Kinetic-pharmacodynamic model to predict post-rituximab B-cell repletion as a predictor of relapse in pediatric idiopathic nephrotic syndrome

**DOI:** 10.3389/fphar.2024.1526936

**Published:** 2025-01-07

**Authors:** Ziwei Li, Qian Shen, Hong Xu, Zhiping Li

**Affiliations:** ^1^ Department of Pharmacy, Children’s Hospital of Fudan University, National Children’s Medical Center, Shanghai, China; ^2^ Department of Nephrology, Children’s Hospital of Fudan University, National Children’s Medical Center, Shanghai, China

**Keywords:** rituximab, idiopathic nephrotic syndrome, kinetic-pharmacodynamic model, relapse, B-cell

## Abstract

**Purpose:**

Rituximab has proven efficacy in children with idiopathic nephrotic syndrome (INS). However, vast majority of children inevitably experience relapse with B-cell repletion, necessitating repeat course of rituximab, which may increase the risk of adverse effects. The timing of additional dosing and optional dosing regimen of rituximab in pediatric patients with INS have yet to be determined. This study aimed to identify factors that influence disease relapse and B-cell repletion to provide tailored treatment.

**Methods:**

LASSO and random survival forest were performed on 143 children to screen covariates which were then included in Cox regression model to determine the biomarkers of relapse and establish a nomogram. A kinetic-pharmacodynamic (K-PD) model was developed in 59 children to characterize the time course of CD19^+^ B-cell after rituximab treatment. Monte Carlo simulation was conducted to explore a mini-dose regimen with larger intervals.

**Results:**

Nomogram contained 7 predictors of relapse including neutrophil-to-lymphocyte ratio, duration of B-cell depletion, duration of disease, urine immunoglobulin G to creatinine ratio, urine transferrin, duration of maintenance immunosuppressant and hemoglobin. As a direct PD indicator, each 1-month increase of duration of B-cell depletion decreased risk of relapse by 21.4% (HR = 0.786; 95% CI: 0.635–0.972; *p* = 0.026). The K-PD model predicted t_1/2_ (CV%) of rituximab and CD19^+^ B-cell to be 11.6 days (17%) and 173.3 days (22%), respectively. Immunoglobulin A is an important covariate of ED_50_. Simulation of a mini-dose regimen with larger intervals (three 150 mg every 2 monthly) indicted longer B-cell depletion time (>7 months) compared to standard regimen.

**Conclusion:**

The nomogram indicated optimal infusion timing before relapse and the K-PD model provided tailored rituximab regimens for children with INS to reduce safety risks and financial burden.

## 1 Introduction

Idiopathic nephrotic syndrome (INS), characterized by heavy proteinuria, hypoalbuminemia, edema and hyperlipidemia, is the most common glomerular disease in children (Trautmann et al., 2023). Although most cases are steroid sensitive NS and respond well to steroids therapy, up to 50% of children develop frequent relapsing or steroid dependent NS (FRSDNS), requiring addition of steroid-sparing immunosuppressants (Trautmann et al., 2023; Webb et al., 2019). Among the children who do not respond, defined as having steroid-resistant NS, most respond to immunosuppressants, mainly with calcineurin inhibitors, while children in whom no response is observed are described as multi drug resistant. It has been demonstrated that overexpression of P-glycoprotein on lymphocytes result in poor response and steroid resistance (Prasad et al., 2015). The changes in histological appearance to focal segmental glomerulosclerosis are associated with multi drug resistance. Also circulating factors are present in multi-drug-resistant NS (Vivarelli et al., 2023; Ying et al., 2021). Rituximab, a B-cell-targeting anti-CD20 monoclonal antibody, has emerged as an effective treatment for complicated FRSDNS to induce prolonged remission and avoid steroids toxicities, allowing withdrawal of steroids and concomitant immunosuppressants (Chan et al., 2023; Chan et al., 2022). Clinical efficacy of rituximab depends on B-cell depletion, and responders are prone to relapse following B-cell repletion (Colucci et al., 2016). 80% of children eventually relapse 1 year after rituximab (Chan et al., 2020), necessitating either repeat course of rituximab or addition of steroid-sparing immunosuppressants (Chan and Tullus, 2021). Despite its excellent efficacy and favorable safety profile, repeat courses of rituximab may increase the risk of adverse effects, including infusion reactions and hypogammaglobulinemia, especially in young children (Sinha et al., 2023). However, the timing of additional dosing and optional dosing regimen of rituximab in pediatric patients with INS have yet to be determined. The current dosing regimen is based on the treatment of tumor B-cells, which proliferate much more rapidly than normal B-cells in INS. A study in healthy volunteers demonstrated that 97% depletion of circulating B-cell after infusion of 1 mg/m^2^ rituximab, although transient, made it conceivable that lower dose than the authorized (375 mg/m^2^) might be sufficient to deplete all B-cell (Schoergenhofer et al., 2018). Recently, a retrospective study in primary membranous nephropathy (PMN) revealed that monthly mini-dose (100 mg) achieved comparable clinical efficacy with reduced risks and financial burden compared with the standard regimen (Wang et al., 2023). The kinetic-pharmacodynamic (K-PD) model is a powerful tool to quantitative dose-response relationship and accurately predicting the clinical efficacy of different regimens in the absence of PK data (Pan et al., 2019). To our knowledge, no K-PD studies reporting the use of rituximab in pediatric patients with INS.

Therefore, we aimed (1) to identify predictor of relapse with special emphasis the relation with CD19^+^ B-cell to guide re-dosing of rituximab; (2) to investigate covariates associated with efficacy and explore the impact of different dosing regimens on CD19^+^ B-cell repletion time.

## 2 Methods

### 2.1 Patients

We retrospectively collected clinical data of 143 children diagnosed with INS treated with rituximab in Department of Nephrology of Children’ Hospital of Fudan University between January 2014 and December 2022. Inclusion criteria were age <18 years; the first use of rituximab; and at least 6 months of follow-up post-rituximab. Exclusion criteria were congenital or secondary NS [e.g., lupus nephritis, IgA nephropathy, Henoch-Schonlein purpura nephritis (HSPN)], chronic infections such as tuberculosis, HIV, hepatitis B or C and malignancy. CD19^+^ B-cell counts from 59 children were used to develop a K-PD model of rituximab.

### 2.2 Treatment regimen

Rituximab (MabThera^®^ and Henlius^®^, Shanghai, China) course comprised 2 infusions (375 mg/m^2^, maximum 500 mg) given a week apart after attainment of remission. To prevent infusion reactions, patients received methylprednisolone, acetaminophen and antiallergy medications, approximately 30 min before rituximab treatment. Steroids and concomitant immunosuppressive agents were tapered and discontinued within 3–6 months post-rituximab treatment. Mycophenolate mofetil (MMF) was used to maintain remission after B-cell repletion and its protocol and monitoring were individualized. The initial dose of MMF was 20–30 mg/kg/d and subsequently adjusted to an AUC_0–12h_ levels of 30–60 mg·h/L.

### 2.3 Data collection

Clinical and laboratory variables were routinely assessed and reviewed from the medical records to identify biomarkers associated with relapse (baseline at relapse) and B-cell repletion (baseline at rituximab treatment), including demographics, renal histology, steroid responsiveness, age at onset, age at rituximab treatment, duration of disease, prior and maintenance immunosuppressants, hematological and biochemical parameters, immunological profile and urine protein panel.

### 2.4 Outcome

The first outcome was the predictors of relapse with special emphasis the relation with B-cell. Patients who were lost to follow-up or did not relapse were censored at the last follow-up. Remission was defined as urine protein-to-creatinine ratio (UPCR) <0.2 mg/mg or urine dipstick nil or trace for 3 consecutive days. Relapse was defined as UPCR >2.0 mg/mg or urine dipstick ≥3 + for 3 consecutive days (Trautmann et al., 2023).

The second outcome was covariates associated with B-cell repletion and the time course of CD19^+^ B-cell under different rituximab dosing regimen. The duration of B-cell depletion was defined as the time from the rituximab treatment to the first repletion of CD19^+^ B-cell in the peripheral blood. B-cell depletion and repletion were defined as CD19^+^ B-cell <1% and >1% (10/μL) of the total lymphocyte population, respectively (Chan et al., 2022).

### 2.5 Model construction and evaluation

#### 2.5.1 Cox regression model to predict relapse

Addressing multicollinearity and the overfitting problem in high-dimensional data, least absolute shrinkage and selection operator (LASSO) and random survival forests (RSF) were adopted to select significant variables. Lasso regression was a penalized linear regression models in which the coefficients of variables that did not significantly contribute to model performance were shrunk to zero by imposing a shrinkage parameter lambda (λ). The optimal λ was selected the value that yielded the minimum deviance plus one standard deviation in a ten-fold cross-validation process. Features with non-zero coefficients in the LASSO regression with the optimal λ value were retained for subsequent modeling (Lee et al., 2022; Adamichou et al., 2021). RSF algorithms, a non-linear method, was an ensemble of binary decision trees. For each decision trees 1,000 bootstrap sample are randomly drawn from original data, which includes 63% of the observations and remains the left 37% as the out-of-bag data that is used to calculate the prediction error rate. The top 10 optimal variables were selected based on minimum depth and variable importance (VIMP) ranking (Dietrich et al., 2016). The overlapping variables of the two algorithms were entered into Cox regression model to screen core predictive variables and estimate relative risks. Receiver operator characteristic (ROC) curves were performed to evaluate the diagnostic accuracy of predictors of relapse. Kaplan-Meier curves were used to describe relapse-free survival. Restricted cubic spline (RCS) was used to visualize the association of identified variables with relapse risk on a continuous scale. Model performance was assessed using ROC curve, calibration curve, and decision curve analysis (DCA). To facilitate the clinical service, we converted the complex mathematical model into a nomogram.

#### 2.5.2 K-PD model of rituximab to optimize dosing regimen

The time course of CD19^+^ B-cell was described with a turn-over model where the balance between synthesis and degradation rate was disrupted by rituximab, increasing the latter process (Supplementary Figure S1). Structural model was expressed as follows:

$$\frac{dA_{1}}{dt}=-K_{e}\times A_{1},$$

$$\frac{dE}{dt}=K_{in}-K_{out}\times \left(1+E_{\max}\times \frac{{A_{1}}^{\gamma }}{\left({A_{1}}^{\gamma }+{{ED}_{50}}^{\gamma }\right)}\right)\times E,$$

where A_1_ was initial dose of rituximab, K_e_ was a first-order elimination rate constant of rituximab, K_in_ and K_out_ were the zero and first-order rate constants of synthesis and degradation, respectively. E_max_ was the maximum effect of rituximab, ED_50_ was dose of rituximab to achieve 50% of the E_max_, E was CD19^+^ B-cell. The baseline E = K_in_/K_out_. γ was the Hill coefficient, influencing the shape of curve.

Residual variability was the difference between observed and predicted values. Proportional, additive and mixed additive-proportional residual models were tested. The nonlinear mixed effects modeling approach was used to obtain the population typical values of PK parameters and to identify and quantify the covariates that affect the PK parameters. Inter-individual variability was modelled exponentially as follows:

$$\theta_{i}=\theta_{pop}\times {\left(\frac{{covariate}_{i}}{{covariate}_{median}}\right)}^{\beta }\times \exp\;\left(\eta_{i}\right),$$

where θ_i_ was individual parameter, θ_pop_ was typical value of parameter, η_i_ was inter-individual variability, that was the deviation of η_i_ from θ_pop_, which was normally distributed with mean zero and variance of ω^2^, and β quantified the influence of covariate.

Covariates were tested for their effects on K-PD parameters using a stepwise method with forward inclusion (reduction in objective function value (ΔOFV) by 6.63; *p* < 0.01) and backward exclusion (ΔOFV = 10.8; *p* < 0.001) procedure. The model was evaluated by goodness-of-fit plots including the observed versus population and individual predicted values, conditional weighted residuals versus predicted values and time after dose. The visual predictive check (VPC) (1,000 simulations) and bootstrap (500 resamples) were used to evaluate the predictive performance of final model. To quantify the influence of covariates on K-PD parameters, the final model was used to simulate the time course of CD19^+^ B-cell at covariate of maximum, median and minimum of population in the original dataset. Monte Carlo simulations (1,000 replicates) were conducted for standard regimen and mini-dose regimen with larger intervals to explore new dosing regimen.

### 2.6 Statistical analysis

Descriptive statistics were summarized using mean ± SD or median [interquartile ranges (IQR)] for continuous variables and frequencies with percentages [n (%)] for categorical variables. Differences in clinical characteristic between patients with and without relapse were compared with *t*-test or Mann-Whitney *U* test for continuous variables and χ^2^ or Fisher’ exact tests for categorical data. Statistical significance was defined as a two-tailed *p* < 0.05. Analysis was performed using IBM SPSS Statistics (version 27.0) and R software (version 4.2.1). The K-PD model was constructed using the software Phoenix NLME (version 8.4). OriginPro 2021 software (version 9.8.0.200) was used for figures.

## 3 Results

### 3.1 Patients

This study included 143 children (109 males). During the follow-up period of 16.7 (10.5–25.2) months, 83 (58.0%) patients relapsed, with median time to relapse of 14.0 (8.6–21.2) months. The median age at onset and at time of rituximab treatment was 4.0 (2.3–7.5) and 7.9 (4.9–10.6) years, respectively. The median duration of disease was 2.2 (1.1–3.8) years. The median duration of maintenance immunosuppressants (DmIS) was 10.0 (3.0–16.0) months. The mean duration of B-cell depletion was 5.5 ± 1.2 months, with large individual variability. Baseline demographical and clinical characteristics were described in Supplementary Table S1.

### 3.2 Cox regression model to predict relapse

#### 3.2.1 Identification of predictors of relapse

**FIGURE 1:**
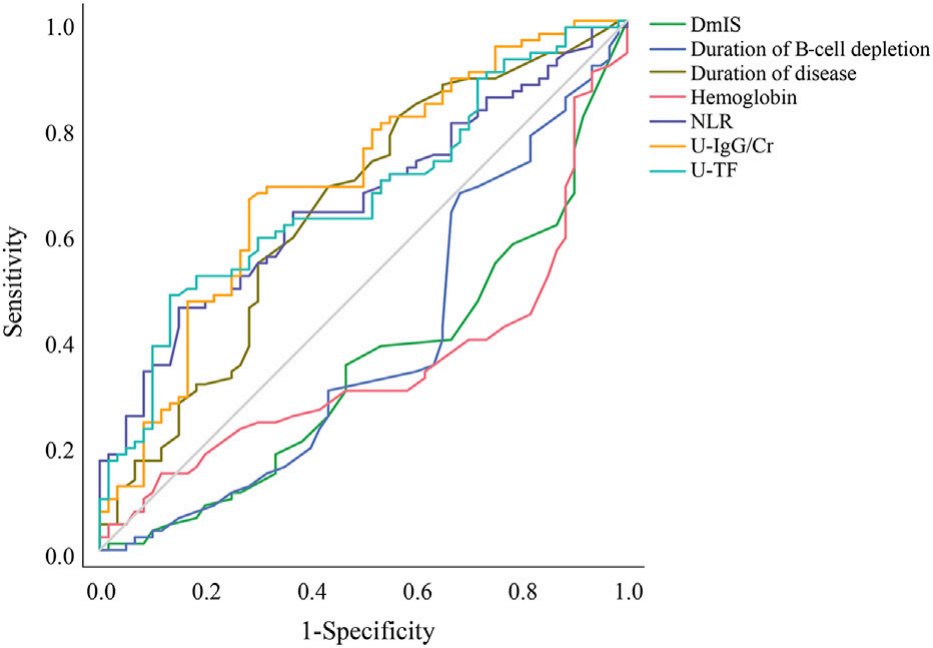
Receiver operating characteristic curves for the diagnostic accuracy of predictors of relapse. DmIS, duration of maintenance immunosuppression; NLR, neutrophil to lymphocyte ratio; U-IgG/Cr, urine immunoglobulin G to creatinine ratio; U-TF, urine transferrin.

The LASSO regression had excellent performance but minimum number of variables when lambda.1se was 0.133 (Supplementary Figure S2). A total of 12 variables were screened out from 46 candidate variables, including neutrophil-to-lymphocyte ratio (NLR), duration of B-cell depletion, urine immunoglobulin G to creatinine ratio (U-IgG/Cr), urine transferrin (U-TF), duration of disease, DmIS, hemoglobin, albumin, alanine aminotransferase, triglyceride, CD4^+^ T cell count and prior immunosuppressants. Of RSF, prediction error rate was minimum when the number of trees was 70. The top 10 important variables were selected according to VIMP (Supplementary Figure S3A) and minimal depth (Supplementary Figure S3B), in the order of DmIS, duration of disease, U-IgG/Cr, hemoglobin, NLR, duration of B-cell depletion, CD16^+^CD56^+^, CD19^+^ %, U-TF and 24-h proteinuria. Seven overlapping predictive variables were input into multivariate Cox regression model, all of which remained significant (Table 1). The correlation coefficients among all variables were <0.5 (Supplementary Figure S4). Duration of disease, NLR, U-IgG/Cr and U-TF were positively correlated with relapse, while DmIS, duration of B-cell repletion and hemoglobin were negatively correlated with relapse. In cox regression model, NLR had the greatest effect on relapse, followed by duration of B-cell depletion. Each 1-unit increase of NLR was associated with a 26.5% increased risk of relapse (HR = 1.265; 95% CI: 1.080–1.481; *p* = 0.003), while each 1-month increase of duration of B-cell depletion was associated with a 21.4% decreased risk of relapse (HR = 0.786; 95% CI: 0.635–0.972; *p* = 0.026). In addition, ROC curves were performed to compare the diagnostic performance of identified variables on relapse (Supplementary Table S2). Among them, U-IgG/Cr showed the best performance in diagnosing relapse (AUC = 0.693; 95% CI: 0.605–0.781; *p* < 0.001), followed by U-TF (AUC = 0.667; 95% CI: 0.578–0.775; *p* = 0.001). The ROC analysis showed that U-IgG/Cr > 70.5 mg/g had a good prevention for relapse with 66.3% sensitivity and 71.7% specificity, while U-TF threshold of 376.0 mg/L had a low sensitivity of 48.2% but a high specificity of 86.7% for relapse. In brief, the ROC (Figure 1) and Kaplan-Meier curves (Supplementary Figure S5) illustrated that the final model could stratify relapse patients well according to identified variable.

**TABLE 1:** Multivariate Cox regression model showing the association of variables with relapse.

Variables	HR	95% CI	*p*-Value
Duration of disease (years)	1.132	1.052–1.217	<0.001
DmIS (years)	0.897	0.870–0.924	<0.001
Duration of B-cell depletion (months)	0.786	0.635–0.972	0.026
Hemoglobin (g/L)	0.964	0.949–0.980	<0.001
NLR	1.265	1.080–1.481	0.003
U-IgG/Cr (mg/g)	1.050	1.012–1.088	0.009
U-TF (mg/L)	1.081	1.011–1.156	0.023

DmIS, duration of maintenance immunosuppression; NLR, neutrophil to lymphocyte ratio; U-IgG/Cr, urine immunoglobulin G to creatinine ratio; U-TF, urine transferrin.

#### 3.2.2 Visualization of relationship between variables and relapse

**FIGURE 2:**
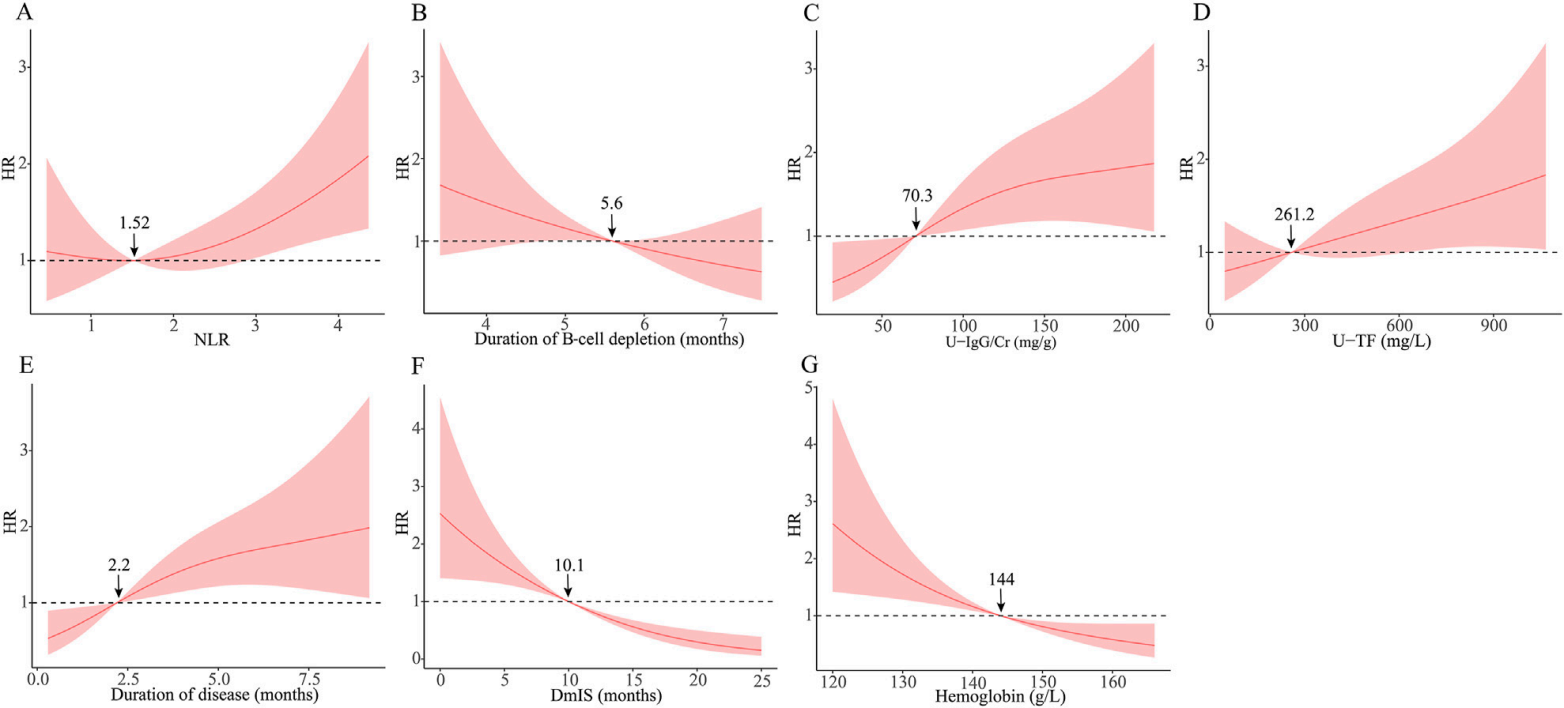
Association between identified variables and relapse risk using restricted cubic spline. The solid line is adjusted HR, the shaded area showing 95% confidence intervals. Reference lines is set at a hazard ratio (HR) of 1.0. DmIS, duration of maintenance immunosuppression; NLR, neutrophil to lymphocyte ratio; U-IgG/Cr, urine immunoglobulin G to creatinine ratio; U-TF, urine transferrin.

RCS was used to visualize the association of identified variables with relapse risk on a continuous scale (Figure 2). The RCS showed a positive linear association between duration of B-cell depletion and relapse risk with a cutoff of 5.6 months, but a negative linear association between U-TF and relapse risk with a cutoff of 261.2 mg/L. The relapse risk decreased with longer DmIS and higher hemoglobin levels but increased with longer duration of disease and higher U-IgG/Cr level, then reached a plateau. The association between NLR and relapse risk was J shaped, and NLR level >1.52 was associated with an increased relapse risk. The cutoff values of identified variables in RCS model were consistent with those in ROC analysis (Supplementary Table S2), except for U-TF.

#### 3.2.3 Model evaluation and validation

For model discrimination, the AUC for predicting relapse risk at 1-, 2- and 3-year were 0.952 (95% CI: 0.918–0.986), 0.906 (95% CI: 0.851–0.961) and 0.931 (95% CI: 0.861–1.00), respectively (Supplementary Figures S6A–C). For model accuracy, the calibration curves for predicting relapse risk at 1-, 2- and 3-year showed a good consistency between the predicted and observed values (Supplementary Figures S6D–F). The clinical net benefit at 1-, 2- and 3-year were above the two extreme curves in DCA curve, suggesting that the model had better clinical predictive efficacy (Supplementary Figures S6G–I). In all, ROC, calibration and DCA curves indicated that the Cox model had good predictive performance.

#### 3.2.4 Nomogram for predicting individual relapse events

**FIGURE 3:**
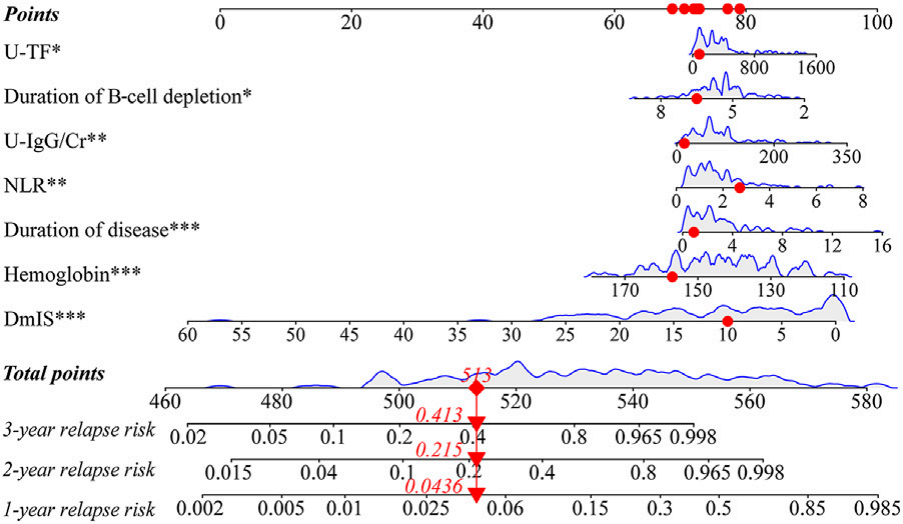
Nomogram for predicting 1-, 2-, and 3-year relapse risk. Red dots represented that a patient with a duration of disease of 0.9 months, hemoglobin of 156 g/L, NLR of 2.72, U-IgG/Cr of 15.5 mg/g, U-TF of 86.2 mg/L, DmIS of 10 months and duration of B-cell depletion of 6.5 months, had a total score of 513, for a predicted 1-, 2- and 3-year relapse risk of 4.4%, 21.5%, and 41.3%, respectively.

For example, a patient with a duration of disease of 0.9 months, hemoglobin of 156 g/L, NLR of 2.72, U-IgG/Cr of 15.5 mg/g, U-TF of 86.2 mg/L, DmIS of 10 months and duration of B-cell depletion of 6.5 months, had a total score of 513, for a predicted 1-, 2- and 3-year relapse risk of 4.4%, 21.5%, and 41.3%, respectively (Figure 3).

### 3.3 K-PD model of rituximab to optimize dosing regimen

#### 3.3.1 Exploration of predictors of B-cell repletion

A total of 526 measurements of CD19^+^ B-cell counts from 59 children were available for K-PD model. Baseline characteristics were summarized in Supplementary Table S3. The baseline IgA level and CD19^+^ B-cell counts were 1.15 ± 0.52 g/L and 727.18 (423.60–964.76)/μL, respectively. The CD19^+^ B-cell counts decreased to <10/μL in all patients following rituximab and gradually recovered within 5.1 ± 1.2 months. The parameter estimates of K-PD model were summarized in Table 2. The elimination rate constants of rituximab (K_e_) and CD19^+^ B-cell (K_out_) suggested t_1/2_ (CV%) of 14.1 (9.9%) days and 99 (11.4%) days, respectively. A mixed additive-proportional residual model best described residual variability. The PD parameter ED_50_ was 1.31 mg. Baseline IgA levels were identified as a significant covariate of ED_50_, explaining 27.4% inter-individual variability.

**TABLE 2:** Parameter estimates from kinetic-pharmacodynamic model and bootstrap.

Parameter	Estimates (RSE%)	Shrinkage (%)	Bootstrap (n = 500) median, 95% CI
Structural parameter			
K_e_ (/day)	0.049 (9.9)	NA	0.049 (0.045–0.067)
K_in_ (/day)	4.14 (10.8)	NA	4.10 (3.32–5.26)
K_out_ (/day)	0.007 (11.4)	NA	0.007 (0.006–0.011)
E_max_	389.7 (15.5)	NA	377.6 (212.2–514.2)
ED_50_ (mg)	1.31 (58.2)	NA	1.21 (0.18–1.79)
γ	6.65 (21.8)	NA	5.78 (2.76–11.58)
Covariate			
IgA on ED_50_	1.39 (17.2)	NA	1.31 (0.74–2.00)
Interindividual variability			
ω_Ke_ (%)	13.4 (15.2)	44.2	14.8 (8.6)
ω_in_ (%)	37.8 (11.6)	21.3	37.2 (9.9)
ω_out_ (%)	24.9 (17.6)	46.4	24.1 (12.4)
ω_Emax_ (%)	57.8 (14.4)	17.5	59.9 (21.5)
ω_ED50_ (%)	113.3 (62.5)	27.6	98.7 (72.6)
Residual variability			
σ_add_ (mg)	2.56 (4.2)	NA	2.41 (0.48–3.08)
σ_prop_ (%)	0.40 (7.6)	NA	0.42 (0.34–0.72)

RSE: residual standard error [RSE=(standard error/estimate)×100]; 95% CI, 95% confidence interval; K_e_, elimination rate constant of rituximab; K_out_, elimination rate constant of CD19^+^.lymphocytes; E_max_, the maximum inhibition effect of rituximab, ED_50_, dose of rituximab to achieve 50% of the E_max_; γ, the Hill coefficient, influencing the shape of curve; IgA, immunoglobulin A; ω, inter-individual variability which was deviation of individual from population; σ, residual variability which was the difference between observed and predicted values, σ_add_, additive residual variability; σ_prop_, proportional residual variability; NA, not applicable.

#### 3.3.2 Model evaluation and validation

**FIGURE 4:**
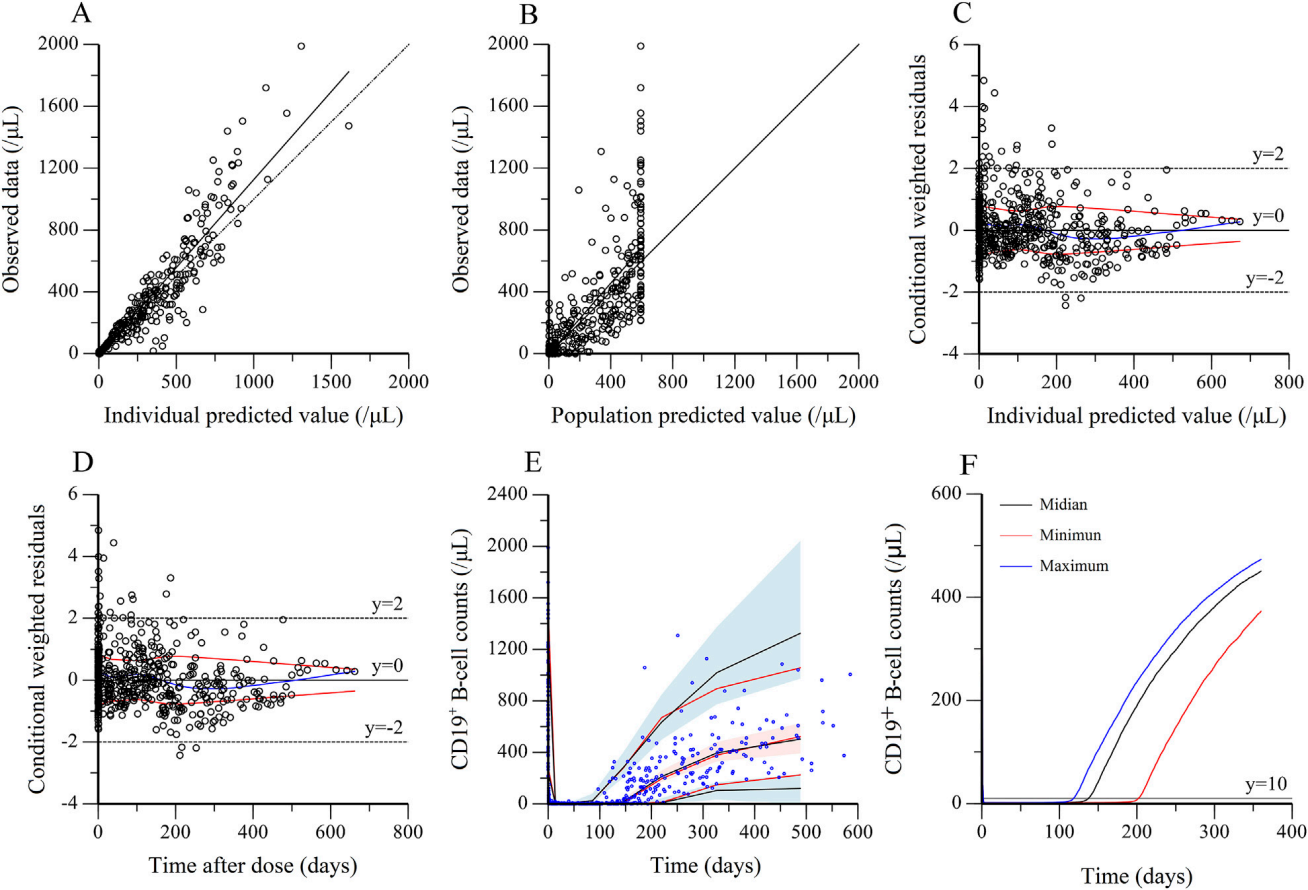
Evaluation and validation of the final kinetic-pharmacodynamic model. **(A)** Observed data versus individual predicted value. **(B)** Observed data versus population predicted value. **(C)** Conditional weighted residuals versus individual predicted. **(D)** Conditional weighted residuals versus time after dose. **(E)** Visual predictive check for prediction accuracy of the final model, in which circles were raw data, the solid red lines represent the 5th, 50th, and 95th percentiles of the observed data. The solid black lines represent the 5th, 50th, and 95th percentiles of the simulated data. The shaded areas represent the 90% confidence interval around the 5th, 50th and 95th percentiles of the simulated data. **(F)** The effect of IgA on B-cell repletion. The simulation is performed by varying IgA level for two weekly 375 mg/m^2^ rituximab treatment. The maximum, median and minimum of IgA were 0.11, 1.15 and 2.26 g/dL, respectively.

The observed values versus individual (Figure 4A) and population predicted values (Figure 4B) were uniformly distributed around the reference line, indicating a good fit of the model, except for a discordance of observed and population predicted values at high values. The conditional weighted residuals versus population predicted values (Figure 4C) and time after dose (Figure 4D) were homogenously distributed within ± 2 unit, confirming that the model had a good stability and accuracy, and without systematic deviation over time, respectively. The population estimations of parameters in the final model were close to median obtained from the bootstrap method and within 95% CI, which demonstrated that the model was stability (Table 2). For VPC, the observed quantiles were consistent with predicted quantiles for the 5th, 50th and 95th percentiles and within the 95% CI, which suggested that the model prediction was accurate (Figure 4E). The time course of CD19^+^ B-cell repletion was simulated at covariate IgA levels at maximum, median and minimum of 0.11, 1.15 and 2.26 g/dL, showing a great effect of IgA on CD19^+^ B-cell repletion (Figure 4F).

#### 3.3.3 Dosing simulation

**FIGURE 5:**
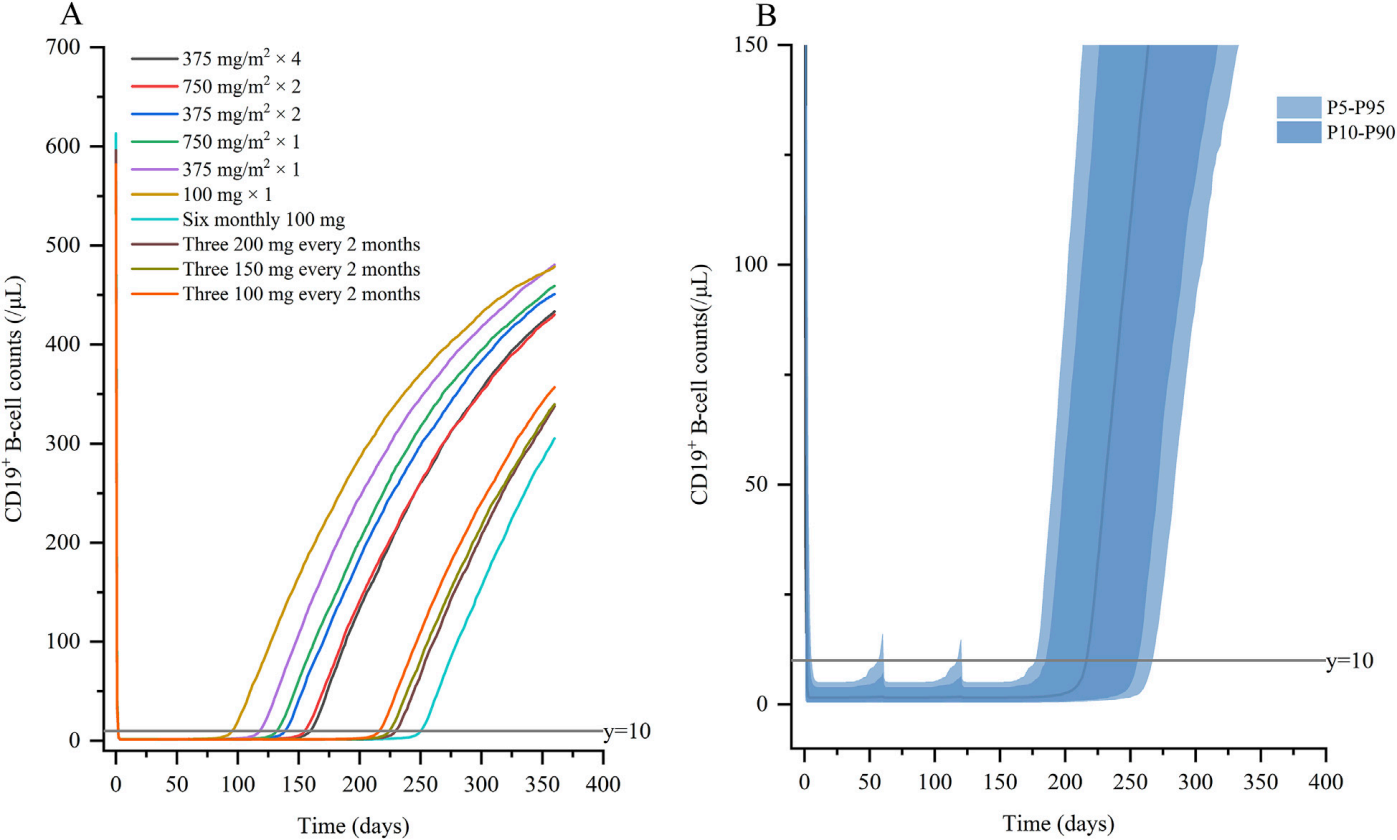
Simulated profiles of suppression of CD19^+^ B-cell **(A)** under variable dosing regimens of rituximab; **(B)** for three 100 mg every 2 months. Percentile bands of simulated data are represented in different shades of blue.

Based on covariate model, we carried out simulations for both standard dose and monthly mini-dose to predict CD19^+^ B-cell counts (Figure 5A; Table 3). For standard regimen, the suppression of rituximab on CD19^+^ B-cell weakened with the decrease of dosing frequency, but not significant. However, the repletion of CD19^+^ B-cell occurred more rapidly with single infusion of 375 mg/m^2^ compared to 4 weekly 375 mg/m^2^. For monthly mini-dose with larger interval, six monthly 100 mg and three 200 mg every 2 monthly can maintained B-cell depletion for more than 8 months, much longer than 2 weekly 375 mg/m^2^. When dose was reduced to three 150 mg every 2 monthly and three 100 mg every 2 monthly, the suppression of B-cell lasted for more than 7 months in 95% and 90% of patients, respectively (Figure 5B). A dose of three 150 mg every two monthly was recommended for children with INS, with lower cumulative dose and safety risks.

**TABLE 3:** Simulated repletion time to 10/μL for patients receiving rituximab.

Dosing regimen	Time to B-cell repletion (90% PI) (day)
375 mg/m^2^ × 4	160 (113∼219)
750 mg/m^2^ × 2	155 (109∼210)
375 mg/m^2^ × 2	139 (95∼196)
750 mg/m^2^ × 1	133 (89∼189)
375 mg/m^2^ × 1	119 (75∼171)
100 mg × 1	96 (55∼146)
Six monthly 100 mg	251 (211∼302)
Three 200 mg every 2 monthly	230 (190∼281)
Three 150 mg every 2 monthly	225 (183∼275)
Three 100 mg every 2 monthly	217 (178∼267)

90% PI: 5%–95% percentile interval.

## 4 Discussion

A nomogram was established for predicting relapse to guide timing of additional dosing, which not only confirmed the relationship between B-cell repletion and relapse, but also identified NLR, U-IgG/Cr and U-TF as predictors in pediatric INS for the first time. Second, a K-PD model was first built in pediatric INS to predict B-cell repletion and explore new dosing regimens.

NLR was positively associated with risk of relapse (HR = 1.265; 95% CI: 1.080–1.481; *p* = 0.003) with a cutoff value of 2.13. Our finding was similarly to two previous studies in patients with IgA nephropathy, one showing NLR <2.43 as an effective predictor of corticosteroid response (HR = 1.252; 95% CI: 1.097–1.407; *p* < 0.001) (Yang et al., 2019), the other showing NLR >2.67 as an independent risk factor for diseases progression (HR = 1.74; 95% CI: 0.98–3.05; *p* = 0.043) (Wang et al., 2021). Furthermore, higher NLR (>2.41) was an independent indicator for poor renal prognosis in IgA vasculitis with nephritis (HR = 2.689; 95% CI: 1.044–6.927; *p* = 0.040) (Li et al., 2022). NLR reflected the balance between inflammatory and immune response. Increased NLR indicated an ongoing nonspecific inflammatory pathway and relatively inadequate immunity status, resulting in decline in ability to resist disease (Dong et al., 2023). Inflammation was the most important trigger of progressive tubulointerstitial fibrosis and renal scarring (Wang et al., 2021).

Another two biomarkers were U-IgG/Cr and U-TF. Massive excretion of high molecular weight protein (IgG) reflected severe disruption of glomerular selectivity permeability, while increased U-TF level was due to decreased tubular reabsorption, both of which were associated with tubulointerstitial inflammation and damage (Bazzi et al., 2001; Branten et al., 2005; Tang et al., 2002). Previous studies in patients with PMN reported that patients with U-IgG/Cr <110 mg/g had higher remission and lower progression to chronic renal failure (CKD) (Bazzi et al., 2001), and that U-IgG >250 mg/24 h was an independent predictor for development of renal insufficiency (Branten et al., 2005). Besides, IgG fractional excretion was a good predictor of CKD progression and responsiveness to immunosuppressants in IgA nephropathy (Bazzi, 2014). Also, U-TF demonstrated an excellent ability to predict active lupus nephritis and response to rituximab (Davies et al., 2021). And a study in children with HSP showed that U-IgG (OR = 1.48; 95% CI: 1.23–1.85; *p* < 0.010) and U-TF (OR = 1.50; 95% CI: 1.17–2.22; *p* = 0.013) were significantly associated with progression of renal involvement (Liu et al., 2019). Of note, PSI, the ratio between the urine clearance of IgG and that of TF, proven to be a good predictor of rituximab response in adult INS (Allinovi et al., 2022).

In clinical practice, depletion and repletion of CD19^+^ B-cell was a pharmacologic marker to follow rituximab response and relapse. An earlier study enrolled 46 children with multi-drug-dependent NS (Ravani et al., 2013), suggested that successful response to rituximab was associated with longer time to CD20 B-cell repletion, which was 185 (95% CI: 104–267) days in children who achieved prednisone and calcineurin-inhibitor-free remission for 6 months versus those who did not [109 (45–172) days; *p* = 0.01]. Another study of 37 children with complicated SDNS, early B-cell repletion (<5 months) was an independent risk factor for early relapse (<12 months) after rituximab treatment (Fujinaga and Hirano, 2014). Recently, a study involving 61 children with SDNS showed one-year relapse-free survival was significantly associated with time to B-cell depletion [HR of relapse 0.78 (0.63–0.97) per month of B-cell depletion] (Hogan et al., 2019). In addition, a retrospective cohort study of children with FRSDNS concluded that longer relapse-free periods were associated with longer B-cell depletion period (>6.0 months, HR = 0.36; 95% CI: 0.22–0.61; *p* < 0.001) (Choi et al., 2024). These findings were consistent with our result, whereby longer B-cell depletion period (>5.7 months; HR = 0.786; 95% CI: 0.635–0.972, *p* = 0.026) had a good prevention for relapse. The efficacy of B-cell-depleting agents (e.g., rituximab) at maintaining long-term remission supported an immune mediated etiology and emphasized a central role for B-cells in INS pathogenesis. Recently, the identification of pathogenic antibodies directed against podocytes, such as anti-nephrin antibodies reinforced the idea of the involvement of B-cells, and in particular antibody-secreting B-cells, in the development of INS. Finally, relapse of INS often coincided with an immunity-triggering event, including respiratory tract infections, gastroenteritis, and allergic reactions, reminding us of the importance of humoral immunity in the development of this disease (Al-Aubodah et al., 2024; Dorval et al., 2024).

Next, we developed a K-PD model including IgA to predict B-cell repletion and to estimate individual dose. We showed that the K-PD model was able to uncover underlying PK information in the absence of rituximab concentrations, especially for pediatric patients with difficulty in blood collection. The t_1/2_ of rituximab (14.1 days) was comparable to that reported in previous study, such as PMN (14.7 days) (Liang et al., 2024), rheumatoid arthritis (17.3 days) (Lioger et al., 2017) and autoimmune diseases (19.3 days) (Pan et al., 2019). Compared to current K-PD models of rituximab in autoimmune diseases (Pan et al., 2019; Riva et al., 2023), the t_1/2_ of CD19^+^ B-cell (99 days) was higher than that reported by the former (35 days), but lower than the latter (173 days), and ED_50_ was similar (1.31 versus 0.81 versus 0.692 mg), while E_max_ were higher (390 versus 35.2 versus 155 mg). Baseline IgA levels as covariate of ED_50_, were further included to improve the model. Patients with higher IgA levels required higher rituximab dose, meaning that those had a shorter time to B-cell repletion at the same dose. Consistent with the results from a retrospective cohort study of 224 patients with autoimmune diseases, higher levels of IgA (HR = 1.21, 95% CI: 1.01–1.45, *p* = 0.040) were positively associated with B-cell repletion (Nie et al., 2023). In addition, two studied in patients with rheumatoid arthritis demonstrated that a negative correlation between baseline IgA levels and time to B-cell repletion (*p* = 0.007), and patients with elevated baseline IgA levels had significantly higher B-cell counts 5 months after rituximab compared to those with normal IgA levels (*p* = 0.04) (De La Torre et al., 2012a; De La Torre et al., 2012b). The association of serum IgA with B-cell kinetics is not so clear, but suggests that microbial dysbiosis or infection-triggered mucosal immunity may play a role. The mucosal immune system was known to primarily consist of lymphoid tissues that were distributed in the skin, oral cavity, respiratory tract, urinary tract, and the gastrointestinal tract. IgA as the first line of defense against bacterial and viral infection, which was predominant at mucosa immunity response (He et al., 2020). Children with INS encountered multiple relapses, at least 50% of which were triggered by infection, the most common of which are respiratory, gastrointestinal and urinary tract infections (Uwaezuoke, 2015; Christian et al., 2022). Dysbiosis of oral or gut microbiota might occur in children with relapsing INS (Kawalec and Kiliś-Pstrusińska, 2022; Ye et al., 2024). It has been demonstrated in patients with IgA nephropathy, microbial dysbiosis or infections prime mucosal B-cell activation, leading to overproduction of IgA (Floege and Feehally, 2016). It has previously been reported that early recovery in serum IgA levels after rituximab was due to rapid replenishment of peripherally circulating IgA^+^ plasmablasts/plasma cells which were derived from mucosal B-cells in response to microbial stimulation, leading to subsequent flares and continuous production of autoantibodies. In autoimmune patients, circulating autoreactive plasmablasts can contribute to systemic autoantibody production (Mei et al., 2010; Kridin and Ahmed, 2020).

Although the off-label use of RTX was widely adopted for INS treatment, the optimal dose was still not known. Based on covariate model, simulation showed that B-cell depletion could last for more than 8 months with six monthly 100 mg doses and three 200 mg every 2 monthly. The cumulative dose of simulation was equal to that in patients with BSA of 0.82 m^2^ (median value of our population) based on two weekly 375 mg/m^2^. We further explored reduced dose and recommend that a minimum dose of three 150 mg every 2 monthly for children with INS. The cumulative dose of three 150 mg every two monthly was reduced by 165 mg compared with two weekly 375 mg/m^2^. According to the price of approximately 2294 RMB per 100 mg rituximab in China, 165 mg rituximab could result in costs of approximately 3785 RMB, which exceeded the average monthly income of Chinese residents (3268 RMB). Three 100 mg every 2 monthly only maintained B-cell depletion for more than 7 months in 90% of children. Children above 90% percentile experienced a transient B-cell repletion before the next dose, which might lead to relapse. This was comparable with previously published papers on rituximab for PMN. One study reported that a single dose of rituximab 100 mg could achieve B-cell depletion in 87.5% of individuals, and this depletion could be maintained for at least 1 month. Monthly rituximab 100 mg appeared as a potential effective regimen for treating PMN (Wang et al., 2023). The other study built a population PK-PD model for rituximab and recommended the novel regimen (6 monthly 100 mg) with the comparable ability and superior duration time of B-cell depletion (>7 months) compared with standard dose (four weekly 375 mg/m^2^), while simulation of a dose of 100 mg every 2 months was insufficient for long time B-cell depletion, supporting our results (Liang et al., 2024). Standard regimen could not fully exploit the potential of rituximab. The maintenance of a minimum level of drug for a prolonged time, seemed to be more important rather than the rapid achievement of a very high dose for a shorter time, meanwhile the lowering rituximab dose would yield reduced medical costs and safety risk.

The main limitation of this study was its retrospective design. However, the inclusion of a large number of pediatric patients with long-term follow-up, were the major strength of this study. Another limitation was the study design did not include histology because it had not been identified as a significant predictor (Chan et al., 2020). Future studies should aim to incorporate histological findings, as they might offer additional predictive value.

## 5 Conclusion

We demonstrated that duration of B-cell depletion was an important predictor of relapse in children with INS and established an accurate nomogram. Then, we built a reliable K-PD model to predict post-rituximab B-cell repletion in the absence of PK concentrations. The nomogram indicated optimal infusion timing before relapse and the K-PD model provided tailored treatment for children with INS to reduce safety risks and financial burden. In future studies, the accuracy of the nomogram and K-PD model need to be verified in clinical practice.

## Data Availability

The original contributions presented in the study are included in the article/Supplementary Material, further inquiries can be directed to the corresponding authors.
