# Supplementary material for: Kinetic-pharmacodynamic model to predict post-rituximab B-cell repletion as a predictor of relapse in pediatric idiopathic nephrotic syndrome
Source: Front Pharmacol. 2025 Jan 7;15:1526936. doi: 10.3389/fphar.2024.1526936 (PMC11746908; doi:10.3389/fphar.2024.1526936)
Supplement: Supplementary file 1 [file DataSheet1.docx]

**Supplementary Materials**

**1 Supplementary Figures**


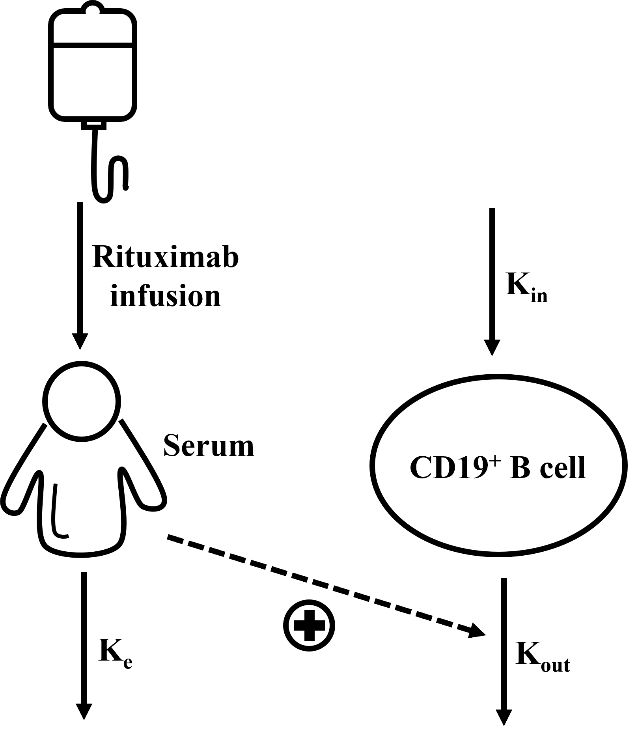


**Supplementary Figure S1.** Diagram of rituximab kinetic-pharmacodynamic model. K_e_, elimination rate constant of rituximab; K_in_, production rate of CD19^+^ B cells; K_out_, elimination rate constant of CD19^+^ B cells; E_max_, the maximum effect of rituximab, ED_50_, dose of rituximab to achieve 50% of the E_max_; plus sign, promotion on CD19^+^ B cells elimination via E(t) =$E_{max}\times{A_{1}}^{\gamma}/({A_{1}}^{\gamma}+{{ED}_{50}}^{\gamma})$; γ, the Hill coefficient, influencing the shape of curve.


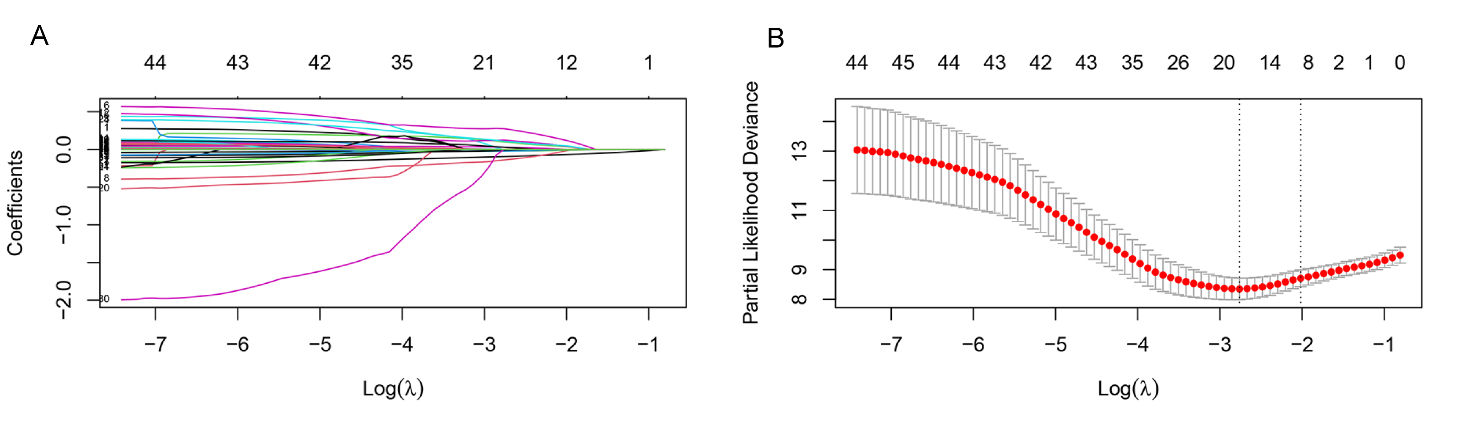


**Supplementary Figure S2.** Variable screening based on LASSO regression. (A) coefficient profiles of 46 candidate variables generated against log lambda (λ) sequence. The coefficients of variables that did not significantly contribute to model performance were shrunk to zero by imposing a shrinkage parameter λ, while variables with non-zero coefficients with the optimal λ value were retained. (B) A ten-fold cross-validation process for the optimal parameter λ which yielded the minimum deviance plus one standard deviation.


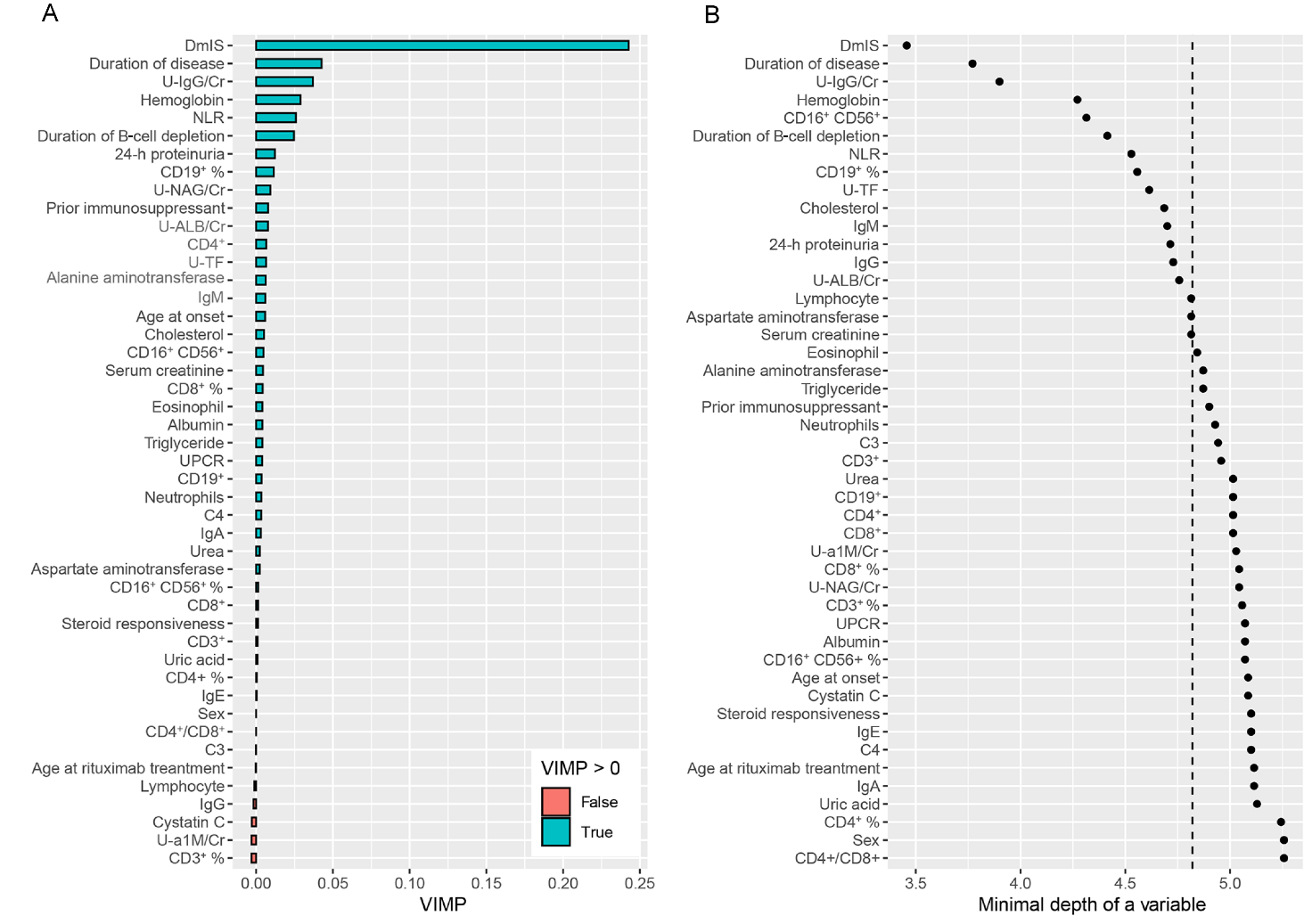


**Supplementary Figure S3.** Variable screening based on random survival forest. The top 10 optimal variables were selected based on (A) variable importance (VIMP) and (B) minimal depth method. DmIS, duration of maintenance immunosuppression; U-IgG/Cr, urine immunoglobulin G to creatinine ratio; NLR, neutrophil to lymphocyte ratio; U-NAG/Cr, urine N-acetyl-β-D-glucosaminidase to creatinine ratio; U-ALB/Cr, urine albumin to creatinine ratio; U-TF, urine transferrin; UPCR, urine protein to creatinine ratio; U-α1M/Cr, urine alpha-1-microglobulin to creatinine ratio.


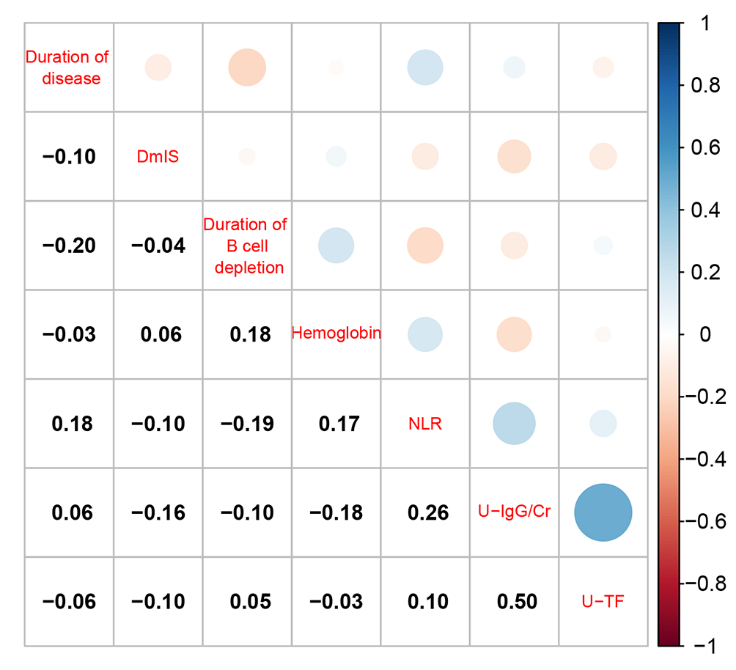


**Supplementary Figure S4.** Correlation matrix of identified variables. DmIS, duration of maintenance immunosuppression; NLR, neutrophil to lymphocyte ratio; U-IgG/Cr, urine immunoglobulin G to creatinine ratio; U-TF, urine transferrin.


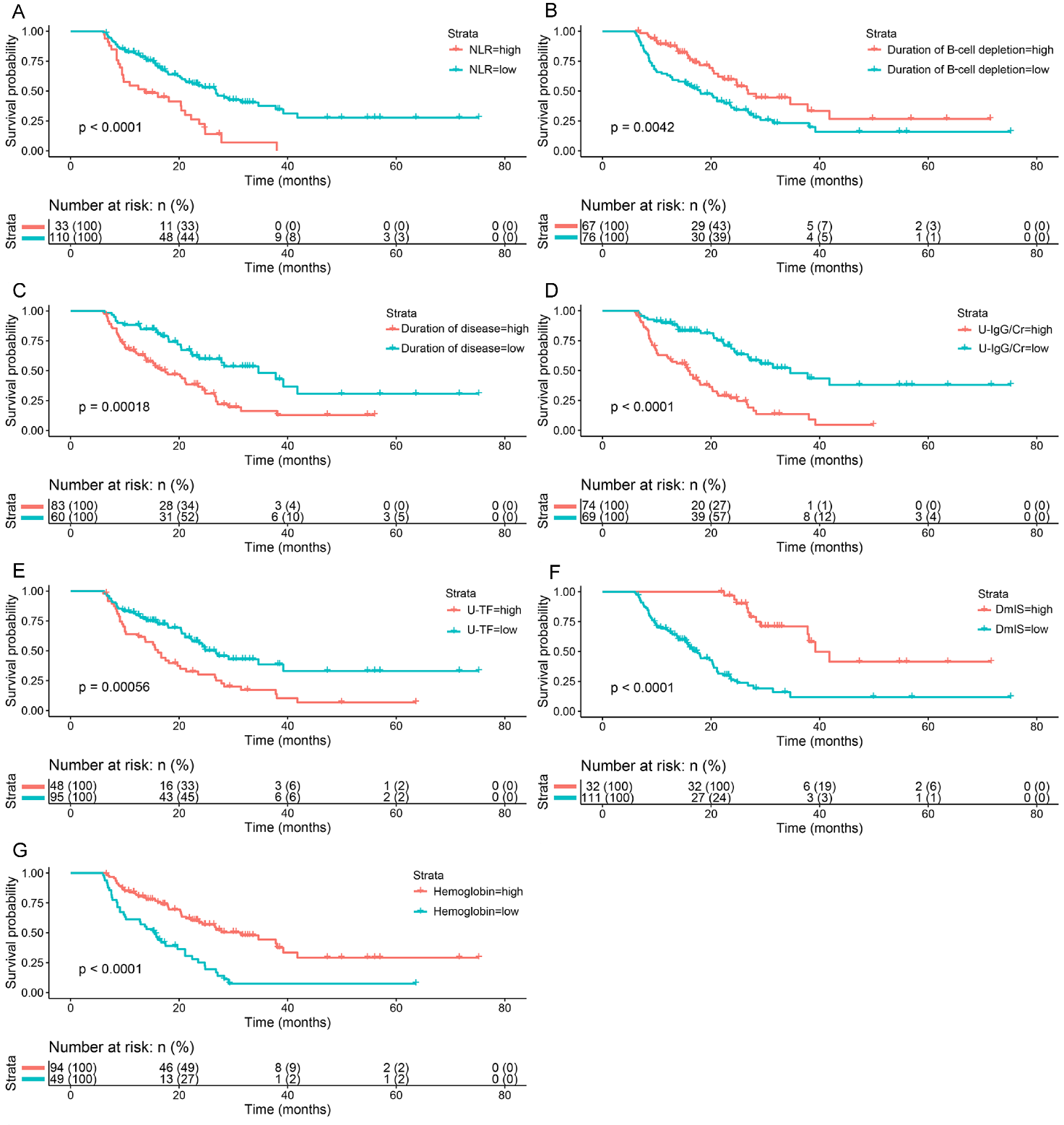


**Supplementary Figure S5.** Kaplan-Meier for survival analysis stratified by identified variables. DmIS, duration of maintenance immunosuppression; NLR, neutrophil to lymphocyte ratio; U-IgG/Cr, urine immunoglobulin G to creatinine ratio; U-TF, urine transferrin.


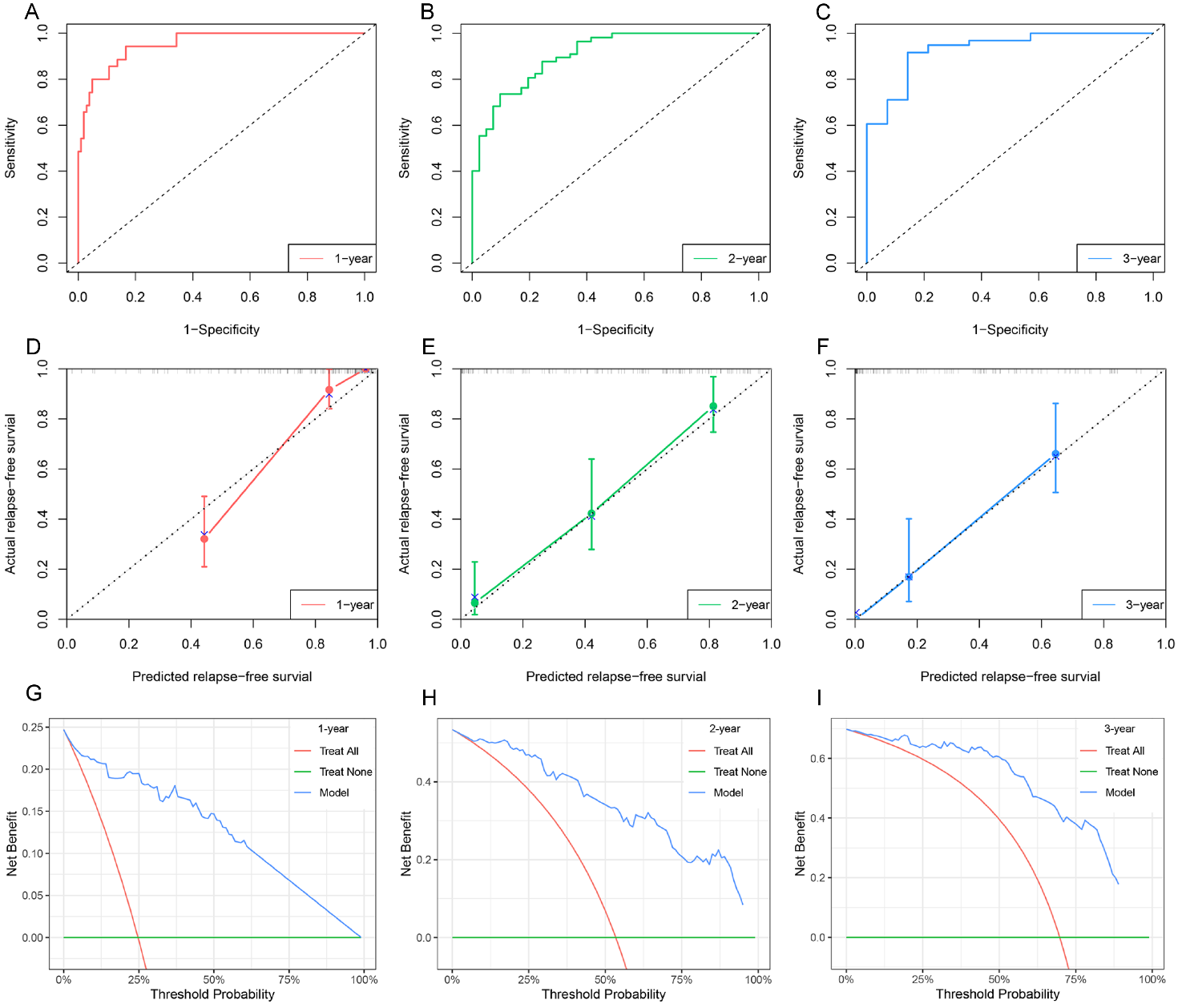


**Supplementary Figure S6.** Evaluation of prediction performance of Cox regression model. (A-C) ROC curve for predicting 1-, 2-, and 3-year relapse-free survival, indicating a good discrimination; (D-F) Calibration curves for comparing predicted and actual 1-, 2-, and 3-year relapse-free survival, indicating a good accuracy; (G-I) Decision curve of the clinical benefit for 1-, 2-, and 3-year relapse-free survival, indicating a better clinical predictive efficacy.

**2 Supplementary Tables**

**Supplementary Table S1.** Baseline characteristics of patients stratified by relapse and non-relapse.

| **Variables** | **All**  **(n=143)** | **Relapse**  **(n=83)** | **Non-relapse**  **(n=60)** | **P value** |
| --- | --- | --- | --- | --- |
| **Demographics** | | | | |
| Sex (female/male) | 34/109 | 66 (79.5%) | 43 (71.7%) | 0.276 |
| Age at onset (years) | 4.0 (2.3-7.5) | 3.5 (2.3-6.8) | 4.1 (2.7-8.7) | 0.229 |
| Age at rituximab treatment (years) | 7.9 (4.9-10.6) | 8.1 (4.9-10.6) | 7.0 (4.8-10.3) | 0.518 |
| **Disease status** | | | | |
| Duration of disease (years) | 2.2 (1.1-3.8) | 2.6 (1.5-4.1) | 1.8 (0.6-3.5) | 0.003 |
| Steroid responsiveness (SRNS) | 51 (35.7%) | 32 (38.6%) | 19 (31.7%) | 0.396 |
| PriorIS | 89 (62.2%) | 56 (67.5%) | 33 (55.0%) | 0.129 |
| DmIS (months) | 10.0 (3.0-16.0) | 7.0 (2.0-15.0) | 11.0 (6.3-20.5) | 0.002 |
| Duration of B-cell depletion (months) | 5.5±1.2 | 5.3±1.1 | 5.8±1.3 | 0.018 |
| **Hematological and biochemical parameters** | | | | |
| Hemoglobin (g/L) | 144±15 | 141±16 | 148±12 | 0.005 |
| Neutrophils (%) | 56.4±16.8 | 58.8±16.8 | 53.1±16.4 | 0.045 |
| Lymphocyte (%) | 34.9±15.1 | 32.6±15.2 | 38.2±14.5 | 0.030 |
| NLR | 1.52 (1.00-2.31) | 1.76 (1.07-2.80) | 1.36 (0.84-1.80) | 0.001 |
| Eosinophil (%) | 0.80 (0.20-2.10) | 0.80 (0.10-2.30) | 0.80 (0.20-1.98) | 0.683 |
| Albumin (g/L) | 23.3 (18.0-31.8) | 20.6 (17.4-29.3) | 27.4 (19.0-34.2) | 0.013 |
| ALT (IU/L) | 11.9 (8.4-16.4) | 12.0 (8.0-18.9) | 11.8 (9.0-14.4) | 0.410 |
| AST (IU/L) | 23.0 (18.6-28.0) | 24.3 (19.0-29.0) | 22.0 (17.3-27.9) | 0.330 |
| SCr (μmol/L) | 37.0 (30.0-45.0) | 37.4 (30.0-43.0) | 36.0 (29.6-48.2) | 0.954 |
| Cystatin C (mg/L) | 0.82 (0.72-0.96) | 0.85 (0.73-1.03) | 0.79 (0.66-0.91) | 0.036 |
| Urea (mmol/L) | 4.40 (3.50-5.10) | 4.40 (3.46-5.10) | 4.24 (3.50-5.25) | 0.966 |
| Uric acid (μmol/L) | 362 (283-438) | 373 (286-448) | 358 (280-435) | 0.581 |
| Triglyceride (mmol/L) | 2.38 (1.57-3.44) | 2.51 (1.80-3.78) | 1.98 (1.40-2.74) | 0.001 |
| Cholesterol (mmol/L) | 8.40 (6.07-11.59) | 9.40 (6.77-11.86) | 7.31 (5.78-10.53) | 0.013 |
| 24-hour proteinuria (g) | 3.71 (1.79-5.88) | 5.07 (2.07-6.85) | 3.71 (1.53-3.71) | 0.007 |
| UPCR (mg/mg) | 7.44 (3.70-10.64) | 7.99 (4.55-12.03) | 5.83 (3.01-9.47) | 0.021 |
| **Immunological profile** | | | | |
| IgA (g/L) | 1.55±0.69 | 1.57±0.67 | 1.51±0.73 | 0.598 |
| IgG (g/L) | 4.94±2.67 | 4.73±2.65 | 5.23±2.70 | 0.276 |
| IgM (g/L) | 1.74±0.67 | 1.76±0.74 | 1.72±0.58 | 0.692 |
| IgE (kU/L) | 103.14 (38.61-284.28) | 116.15 (29.67-317.21) | 89.86 (42.93-202.18) | 0.656 |
| C3 (g/L) | 1.33±0.26 | 1.34±0.30 | 1.32±0.19 | 0.666 |
| C4 (g/L) | 0.31±0.11 | 0.31±0.11 | 0.31±0.10 | 0.957 |
| CD4^+^/CD8^+^ | 1.39±0.45 | 1.37±0.43 | 1.43±0.48 | 0.466 |
| CD16^+^CD56^+^ (/μL) | 247.03 (140.50-333.71) | 232.31 (132.09-363.02) | 247.87 (169.64-309.13) | 0.750 |
| CD19^+^ (/μL) | 475.34 (304.30-687.81) | 428.43 (279.70-673.92) | 563.60 (382.83-695.52) | 0.009 |
| CD3^+^ (/μL) | 1927.90 (1485.70-2776.90) | 1733.30 (1328.50-2513.80) | 2327.45 (1638.88-3136.85) | 0.002 |
| CD4^+^ (/μL) | 976.91 (761.29-1428.45) | 940.00 (687.78-1224.35) | 1177.21 (860.86-1773.11) | <0.001 |
| CD8^+^ (/μL) | 764.01 (554.80-1060.37) | 725.92 (469.93-972.16) | 886.99 (647.80-1283.10) | 0.007 |
| CD16^+^CD56^+^ (%) | 8.53 (5.99-11.07) | 8.84 (5.84-11.09) | 7.63 (6.01-10.86) | 0.851 |
| CD19^+^ (%) | 16.12 (12.60-20.86) | 15.89 (11.79-21.88) | 16.59 (13.52-20.70) | 0.424 |
| CD3^+^ (%) | 71.11±7.90 | 71.52±7.71 | 70.54±8.20 | 0.464 |
| CD4^+^ (%) | 37.31±7.70 | 37.05±7.35 | 37.67±8.21 | 0.641 |
| CD8^+^ (%) | 27.86±6.43 | 28.15±6.89 | 27.45±5.77 | 0.525 |
| **Urine protein panel** | | | | |
| U-α_1_M/Cr (mg/g) | 23.1 (15.3-28.3) | 25.3 (18.7-30.1) | 20.3 (12.8-28.0) | 0.012 |
| U-ALB/Cr (mg/g) | 5811.5 (2455.4-8689.5) | 6105.1 (3409.0-8692.8) | 4279.5 (1812.5-8490.0) | 0.064 |
| U-NAG/Cr (U/mmol) | 2.98 (1.78-4.21) | 3.43 (2.10-4.60) | 2.59 (1.51-3.58) | 0.005 |
| U-IgG/Cr (mg/g) | 70.6 (46.6-104.0) | 85.5 (62.8-114.3) | 64.6 (32.3-86.6) | <0.001 |
| U-TF (mg/L) | 259.0 (122.0-429.8) | 364.0 (134.0-491.0) | 248.5 (87.0-330.0) | <0.001 |

Data represent as mean ± standard deviation, median (25-75th percentile) or n (%). SRNS: steroid resistance nephrotic syndrome; PriorIS: prior immunosuppressant; DmIS, duration of maintenance immunosuppression; NLR, neutrophil to lymphocyte ratio; AST, aspartate aminotransferase; ALT, alanine aminotransferase; SCr, serum creatinine; UPCR, urine protein to creatinine ratio. U-α_1_M/Cr, urine alpha-1-microglobulin to creatinine ratio; U-ALB/Cr, urine albumin to creatinine ratio; U-NAG/Cr, urine N-acetyl-β-D-glucosaminidase to creatinine ratio; U-IgG/Cr, urine immunoglobulin G to creatinine ratio; U-TF, urine transferrin.

**Supplementary Table S2**. The diagnostic accuracy of various predictors for relapse.

| **Variables** | **AUROC** | **95% CI** | **Sensitivity (%)** | **Specificity (%)** | **Cutoff** | **P value** |
| --- | --- | --- | --- | --- | --- | --- |
| Duration of disease (years) | 0.644 | 0.551-0.737 | 0.687 | 0.567 | 2.0 | 0.003 |
| DmIS (years) | 0.654 | 0.564-0.745 | 0.667 | 0.602 | 9.5 | 0.002 |
| Duration of B-cell depletion (months) | 0.614 | 0.518-0.710 | 0.633 | 0.651 | 5.7 | 0.020 |
| Hemoglobin (g/L) | 0.653 | 0.562-0.745 | 0.817 | 0.554 | 140 | 0.002 |
| NLR | 0.656 | 0.567-0.744 | 0.458 | 0.850 | 2.13 | 0.001 |
| U-IgG/Cr (mg/g) | 0.693 | 0.605-0.781 | 0.663 | 0.717 | 70.5 | <0.001 |
| U-TF (mg/L) | 0.667 | 0.578-0.755 | 0.482 | 0.867 | 376.0 | 0.001 |

AUROC, area under the receiver operator curve; DmIS, duration of maintenance immunosuppression; NLR, neutrophil to lymphocyte ratio; U-IgG/Cr, urine immunoglobulin G to creatinine ratio; U-TF, urine transferrin.

**Supplementary Table S3.** Baseline characteristics of patients in kinetic-pharmacodynamic model

| **Variables** | **Values**  **(n=59)** |
| --- | --- |
| Sex (Female/male) | 15/44 |
| Age at rituximab treatment (years) | 5.5 (3.3-7.8) |
| Duration of B-cell repletion (months) | 5.1±1.2 |
| Body surface area (m^2^) | 0.82 (0.68-1.10) |
| Hemoglobin (g/L) | 140±12 |
| Neutrophils (%) | 56.7±17.0 |
| Lymphocyte (%) | 34.6±16.1 |
| Albumin (g/L) | 35.1 (31.9-39.0) |
| Alanine aminotransferase (IU/L) | 19.9 (12.2-31.9) |
| Cystatin C (mg/L) | 0.76 (0.69-0.83) |
| IgA (g/L) | 1.15±0.52 |
| IgG (g/L) | 4.91±2.11 |
| IgM (g/L) | 1.51±0.66 |
| CD19^+^ (/μL) | 727.18 (423.60-964.76) |
| CD4^+^ (/μL) | 1278.22 (946.39-1657.40) |

Data represent as mean ± standard deviation, median (25th-75th percentile).
